# Structural Determinants of Biologic Recognition Across GPCR Classes

**DOI:** 10.3390/cells15211958

**Published:** 2026-10-28

**Authors:** Bianca Fiorillo, Alessandra Guarracino, Oriana Vitale, Federica Moraca, Bruno Catalanotti

**Affiliations:** Department of Pharmacy, University of Naples “Federico II”, Via D. Montesano, 49, I-80131 Naples, Italy; alessandra.guarracino@unina.it (A.G.); oriana.vitale@unina.it (O.V.); federica.moraca@unina.it (F.M.); brucatal@unina.it (B.C.)

**Keywords:** G protein-coupled receptors (GPCRs), biologic therapeutics, nanobodies, GPCR structure, receptor recognition, allosteric modulation, signaling bias, cryo-EM, monoclonal antibodies, structure-guided drug design

## Abstract

**Highlights:**

**What are the main findings?**

- Comparative structural analysis demonstrates that receptor architecture dictates distinct biologic recognition strategies across G protein-coupled receptor (GPCR) Classes A, B, C, and F.
- Across GPCR classes, biologic recognition is governed by the accessibility and three-dimensional organization of receptor surfaces and by the conformational states stabilized upon binding.

**What are the implications of the main findings?**

- The structural principles identified across GPCR classes establish a unified framework linking receptor architecture with biologic recognition, receptor modulation, and pharmacological outcome.
- This framework provides a conceptual basis for the structure-guided design of next-generation GPCR-targeted biologics with improved selectivity, efficacy, and signaling precision.

**Abstract:**

The rapid increase in experimentally resolved G protein-coupled receptor (GPCR)–biologic complexes has substantially improved our understanding of the molecular basis of receptor recognition and modulation. However, despite the growing number of available structures, a comparative framework integrating the major GPCR classes is still lacking. In this review, we analysed experimentally resolved GPCR–biologic complexes across receptor Classes A, B, C, and F to identify the structural principles governing biologic recognition. Our analysis shows that the architecture of each GPCR class shapes distinct recognition paradigms, while two interdependent structural variables emerge across the superfamily: the accessibility and organization of receptor surfaces and the conformational states associated with these regions. Class A receptors primarily expose compact extracellular surfaces, Class B receptors exploit large extracellular domains that support modular ligand recognition, whereas Class C and Class F receptors rely on structured extracellular domains (Venus Flytrap and cysteine-rich domains, respectively) that enable conformational selection and allosteric regulation. Together, these observations establish a structural framework linking receptor accessibility and conformational dynamics to biologic recognition and pharmacological modulation. This framework provides a conceptual foundation for the rational design of next-generation GPCR-targeted biologics with improved selectivity, efficacy, and functional precision.

## 1. Introduction

The development of biologic therapeutics has expanded modern pharmacology by enabling highly selective modulation of molecular targets that remain difficult or inaccessible for conventional small molecules. Monoclonal antibodies, nanobodies, engineered peptides, aptamers, and other biologic modalities have expanded the therapeutic landscape by recognizing large protein surfaces, transient conformational epitopes, and protein–protein interaction interfaces with high specificity. Their applications range from approved therapeutics and investigational clinical candidates to experimental tools employed as structural chaperones, conformational biosensors, or pharmacological probes. Although biologics have transformed the treatment of cancer, autoimmune diseases, and inflammatory disorders, their application to G protein-coupled receptors (GPCRs) has progressed more slowly owing to the unique structural and dynamic properties of this receptor superfamily [1].

Monoclonal antibodies are commonly generated through immunization or display-based selection, whereas nanobodies, derived from camelid heavy-chain-only antibodies, can be selected from immune or synthetic libraries. Their compact architecture and elongated complementarity-determining region 3 (CDR3) loops facilitate recognition of recessed epitopes and stabilization of specific GPCR conformations [2,3]. Engineered peptides exploit endogenous ligand-binding mechanisms, while nucleic acid aptamers are typically selected through Systematic Evolution of Ligands by EXponential Enrichment (SELEX) [4]. Modular biologics combine distinct recognition or activation components within a single construct [5]. Despite their therapeutic potential, these modalities present different challenges, including limited tissue penetration, delivery constraints, proteolytic or nuclease degradation, rapid clearance, potential immunogenicity, and the need for pharmacokinetic optimization.

GPCRs comprise more than 800 membrane proteins in the human genome and constitute the largest family of receptors involved in cellular signal transduction [6]. Their physiological functions encompass virtually every organ system, including neurotransmission, endocrine regulation, immune surveillance, cardiovascular homeostasis, metabolism, sensory perception, development, and tissue repair [7]. Consequently, GPCRs remain the most exploited family of therapeutic targets, accounting for approximately one-third of all approved drugs and continuing to dominate pharmaceutical pipelines across multiple disease areas [8,9].

GPCRs function as highly dynamic molecular machines that continuously sample inactive, intermediate, and active conformational states to transduce extracellular signals into different physiological responses. Ligand binding shifts the equilibrium among these conformations, thereby regulating the recruitment of intracellular transducers, including heterotrimeric G proteins, G protein-coupled receptor kinases (GRKs), β-arrestins, and numerous signaling partners. GPCR signaling is increasingly viewed as a conformational continuum that extends beyond the classical binary model of inactive and active receptor states [10].

Until recently, structural investigation of GPCRs represented one of the greatest challenges in membrane protein biology. Their intrinsic conformational flexibility, low natural abundance, and instability following detergent extraction severely limited high-resolution structural characterization. Consequently, early models of GPCR pharmacology were largely inferred from biochemical, mutagenesis, and pharmacological studies in the absence of direct structural evidence [11].

Recent advances in receptor engineering, lipidic cubic-phase crystallization, synchrotron X-ray crystallography, and single-particle cryo-electron microscopy (cryo-EM) have transformed GPCR structural biology by enabling visualization of receptors in multiple conformational and signaling states [12].

Among the technological advances that enabled this revolution, nanobodies have played an important role. By selectively recognizing transient receptor conformations, intracellular-binding nanobodies stabilize otherwise short-lived signaling states, enabling high-resolution structural determination of GPCR complexes that would otherwise remain inaccessible [3,13].

Cryo-EM has further expanded the repertoire of experimentally resolved GPCR–biologic complexes, providing high-resolution structures of receptors bound to monoclonal antibodies, extracellular- and intracellular-binding nanobodies, engineered peptides, antibody fragments, bispecific antibodies, and engineered ligands. These complexes reveal residue-level interaction networks, a broad repertoire of recognition mechanisms, including extracellular domain engagement, orthosteric vestibule penetration, intracellular signaling interface recognition, conformational rearrangements associated with receptor activation, and the molecular basis of subtype selectivity, biased signaling, and allosteric modulation [13]. Structural information is now routinely exploited to engineer biologics with improved affinity, selectivity, efficacy, and signaling bias.

**Figure 1:**
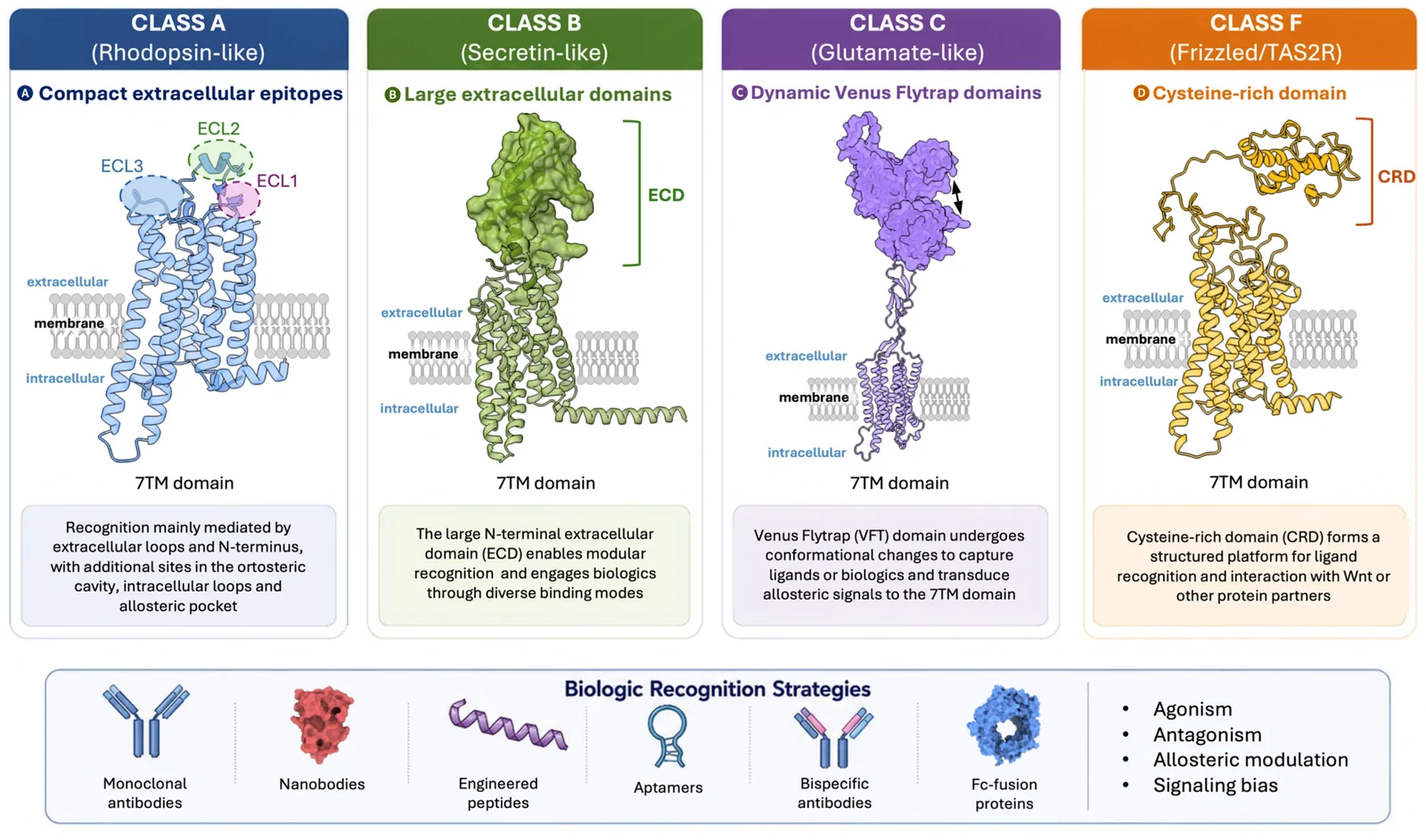
Structural determinants of biologic recognition across G protein-coupled receptor (GPCR) classes. Schematic representation of the four recurrent recognition paradigms employed by biologics to engage GPCRs: (**A**) extracellular surface recognition, (**B**) orthosteric recognition, (**C**) intracellular recognition, and (**D**) allosteric recognition. Despite targeting distinct receptor regions, all strategies converge on conformational stabilization as the common structural principle underlying biologic modulation of GPCR function. Representative receptor structures were generated using AlphaFold models, with the adenosine A2A receptor (A2AR) representing Class A, the glucagon receptor (GCGR) representing Class B, metabotropic glutamate receptor 5 (mGlu5) representing Class C, and Frizzled-5 (FZD5) representing Class F.

The structural diversity of GPCRs is not simply a challenge for biologic discovery but the principal factor governing biologic recognition itself. Differences in extracellular architecture determine epitope accessibility, constrain biologic engagement, and ultimately dictate which recognition mechanisms can be exploited by different biologic modalities (Figure 1). Although all GPCRs share a conserved seven-transmembrane architecture, profound differences in their extracellular organization create distinct recognition landscapes across receptor classes.

Most Class A GPCRs expose relatively restricted extracellular surfaces, limited to the N-terminus and the extracellular loops surrounding the orthosteric vestibule. Thus, for most Class A GPCRs, biologic recognition is directed toward extracellular loops, the orthosteric vestibule, and intracellular signaling interfaces [14,15]. Notable exceptions include glycoprotein hormone receptors, which possess a large leucine-rich repeat extracellular domain, and several chemokine receptors, whose extended N-termini form essential components of protein recognition interfaces.

In contrast, Class B receptors contain large structured extracellular domains that participate directly in peptide recognition. These domains create extensive solvent-accessible protein surfaces well suited for monoclonal antibody binding, explaining why several successful therapeutic antibodies recognize extracellular receptor domains [16].

Class C receptors present an even more distinct recognition landscape through their obligate dimeric organization and characteristic Venus Flytrap domains. Here, biologics frequently modulate receptor activity by stabilizing specific conformations of extracellular domains or dimer interfaces [17].

Finally, Class F receptors rely on extracellular cysteine-rich domains for ligand recognition, creating a recognition landscape distinct from that of the other GPCR classes and expanding the repertoire of biologic interaction mechanisms.

Together, these architectural differences define recurrent recognition paradigms that transcend individual receptors and biologic modalities. Identifying these shared structural principles constitutes the central objective of this review.

The growing number of experimentally resolved GPCR–biologic complexes has generated an important body of structural information. However, most studies remain focused on individual receptors, specific biologic modalities, or isolated therapeutic applications. Consequently, the structural principles shared across GPCR classes are often obscured by receptor-specific descriptions, limiting a broader understanding of how receptor architecture governs biologic recognition.

Several recent reviews have summarized advances in GPCR-targeting antibodies, nanobodies, peptide therapeutics, or individual receptor families [1,3]. However, a comparative structural analysis integrating the major biologic modalities across the principal GPCR classes is still lacking. Such a comparative perspective has become increasingly important as experimentally resolved GPCR–biologic complexes continue to accumulate and biologics move from experimental tools to clinically established therapeutics.

The central premise of this review is that GPCR architecture constitutes the primary determinant of biologic recognition. We examine how receptor architecture shapes epitope accessibility, constrains biologic engagement, stabilizes specific receptor conformations, and ultimately determines pharmacological outcome. By comparing experimentally resolved GPCR–biologic complexes across receptor Classes A, B, C, and F, we identify structural principles shared across the GPCR superfamily while highlighting the recognition paradigms that distinguish individual receptor classes.

The distinctive contribution of this review lies in the integration of structurally characterized GPCR–biologic interactions into a comparative framework extending across Classes A, B, C, and F. By examining recognition mechanisms across receptor architectures and biologic modalities, we identify two interdependent determinants that provide a common basis for interpreting these interactions: the accessibility and three-dimensional organization of receptor surfaces, and the conformational states associated with biologic engagement. This perspective connects residue-level recognition mechanisms with broader principles of receptor selectivity, conformational regulation, and pharmacological activity, providing a framework for interpreting existing structural evidence and guiding future biologic engineering.

Accordingly, the review is organized by major GPCR classes. For each class, we discuss representative GPCR–biologic complexes to identify shared structural features, recurrent binding modes, and common mechanisms of receptor modulation. This class-by-class comparison is intended to define design principles for GPCR-targeted biologics.

## 2. Structural Determinants of Biologic Recognition in Class A GPCRs

Class A GPCRs, also known as the rhodopsin-like receptors, constitute the largest and most pharmacologically relevant branch of the GPCR superfamily, comprising more than 700 members in the human genome [18]. They recognize a wide diversity of endogenous ligands, including biogenic amines, peptides, lipids, nucleotides, chemokines, sensory molecules, and photons, while maintaining a highly conserved seven-transmembrane (7TM) architecture [7].

Although the transmembrane core is structurally conserved, considerable diversity exists within the extracellular loops (ECL1–ECL3), the receptor N-terminus, and the intracellular regions responsible for transducer coupling. These variable structural elements largely determine ligand selectivity, receptor activation, and intracellular signaling specificity. Consequently, they also define the repertoire of epitopes that can be exploited by biologics.

Class A receptors generally lack large extracellular domains, with notable exceptions including glycoprotein hormone receptors and several chemokine receptors. This architectural feature influences biologic recognition. Conventional monoclonal antibodies have relatively limited access to deeply buried orthosteric ligand-binding pockets and therefore predominantly recognize solvent-exposed extracellular regions. In contrast, nanobodies, owing to their compact dimensions and elongated complementarity-determining region 3 (CDR3), can penetrate narrow extracellular vestibules or intracellular cavities that are inaccessible to conventional antibodies [2,3].

Structural studies indicate that biologics recognize Class A GPCRs through several recurrent mechanisms, including extracellular surface recognition, orthosteric pocket penetration, intracellular active-state stabilization, and allosteric modulation. Although each receptor exhibits unique structural features, these recognition modes recur among unrelated Class A receptors, suggesting that they represent conserved structural solutions imposed by the common architecture of the family.

Importantly, these recognition mechanisms are not simply determined by epitope accessibility but are intrinsically coupled to receptor function. Extracellular biologics frequently interfere with endogenous ligand recognition or stabilize inactive receptor conformations, whereas intracellular-binding nanobodies mimic G proteins or arrestins, stabilizing active signaling states. Other biologics recognize extracellular vestibules or allosteric surfaces without directly competing with endogenous ligands, thereby modulating receptor activation through long-range conformational coupling. Together, these observations indicate that biologic recognition in Class A GPCRs is fundamentally a process of conformational selection and stabilization that extends beyond simple molecular binding.

Accordingly, the following sections examine the principal structural determinants governing biologic recognition in Class A GPCRs. Representative structural examples are used to illustrate four major recognition strategies that recur throughout the available structural literature: (i) extracellular surface recognition, (ii) orthosteric pocket penetration, (iii) intracellular active-state stabilization, and (iv) allosteric recognition. Comparative analysis of these paradigms provides the basis for understanding how the conserved architecture of Class A GPCRs shapes biologic engagement and ultimately determines receptor pharmacology.

Although these recognition strategies appear highly diverse, comparative analysis reveals that they originate from a surprisingly limited number of structural determinants imposed by the conserved Class A receptor architecture.

### 2.1. Extracellular Surface Recognition: Exploiting Accessible Epitopes

**Figure 2:**
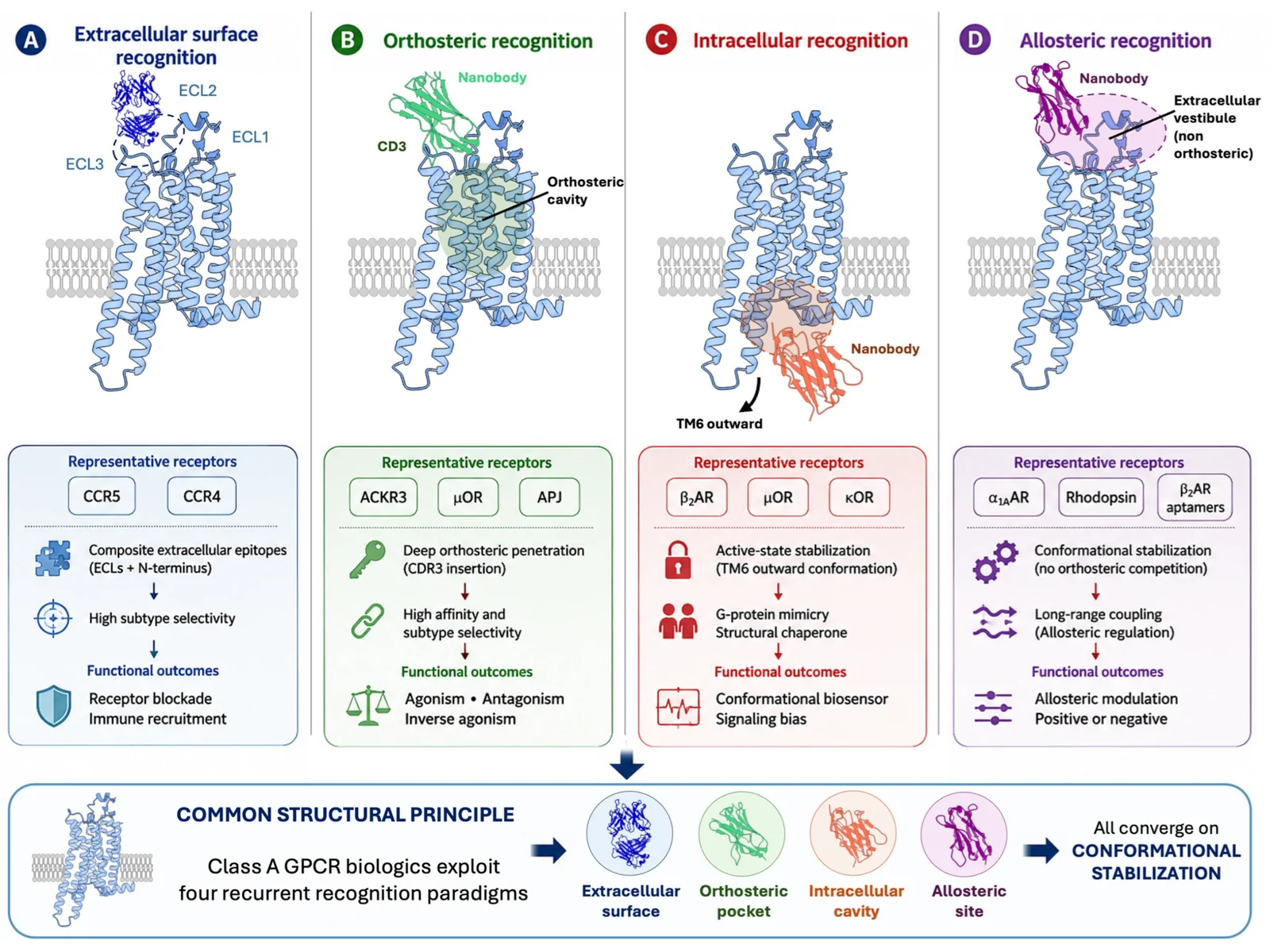
Schematic representation of the four principal recognition strategies currently identified for biologics targeting Class A GPCRs. (**A**) Extracellular surface recognition, involving composite epitopes formed by the receptor N-terminus and extracellular loops; (**B**) orthosteric pocket recognition, mediated by penetration of elongated nanobody complementarity-determining region 3 (CDR3) loops into the ligand-binding cavity; (**C**) intracellular active-state recognition, in which conformational nanobodies occupy the transducer-binding cavity and stabilize active receptor conformations; and (**D**) extracellular allosteric recognition, involving non-orthosteric surfaces coupled to the transmembrane core. The transmembrane receptor shown in all panels corresponds to the AlphaFold-predicted structure of the human adenosine A2A receptor (A2AR), used as a representative Class A GPCR for illustrative purposes. The Fab fragment depicted in (**A**) was derived from PDB ID: 6IDH, whereas the nanobody structures shown in (**B**–**D**) were derived from PDB ID: 7MFV.

The extracellular surface represents the most accessible structural landscape for biologic recognition in Class A GPCRs (Figure 2A).

Among Class A GPCRs, chemokine receptors represent a unique case because they possess relatively long N-terminal segments enriched in acidic and sulfated tyrosine residues that participate directly in chemokine recognition. Therefore, chemokine receptors provide perhaps the clearest example of extracellular surface recognition because their endogenous activation mechanism already relies on extensive extracellular receptor–ligand interactions. According to the classical two-site (chemokine recognition sites 1 and 2, CRS1/CRS2) model, the globular core of the chemokine initially binds to the CRS1 interface at the N-terminal end of the receptor, whereas the chemokine N-terminus penetrates into the transmembrane bundle (CRS2) to activate the receptor through the CRS2 site. This multipartite recognition mechanism naturally creates large extracellular interaction surfaces, suited for biologic targeting.

The crystal structure of CCR5 in complex with macrophage inflammatory protein-1 alpha (MIP-1α) (PDB ID: 7F1T), together with the corresponding cryo-EM structure of the active CCR5-MIP-1α-G*_i_* complex (PDB ID: 7F1Q), provided the first atomic description of this recognition mechanism [19]. The receptor N-terminus (residues Pro8-Glu18) docks into a groove formed by the N-loop, the 40s-loop and the β3-strand of MIP-1α. Three tyrosine residues (Tyr10, Tyr14 and Tyr15), which represent the major sulfation sites of CCR5, are positioned adjacent to the basic residues Arg17, Lys44, Arg45 and Arg47 of the chemokine, establishing the electrostatic framework that defines the CRS1 interaction surface. In addition, a hydrophobic interaction core involving Ile9, Tyr10 and Ile12 of CCR5 and Phe23 of MIP-1α, together with a polar interaction network formed by Asp11, Asn13 and Arg47, further stabilizes the receptor–chemokine complex. Disulfide cross-linking experiments confirmed these residue-level contacts, supporting the structural model proposed by the crystal structure.

These structural observations explain why therapeutic biologics preferentially recognize the extracellular receptor surface with limited access to the orthosteric cavity. The antibody leronlimab (PRO140) binds a conformational epitope spanning the receptor N-terminus and ECL2, thereby preventing the conformational rearrangements required for HIV-1 gp120 engagement while largely preserving physiological chemokine signaling [20].

A similar recognition strategy is observed for CCR4, where the afucosylated monoclonal antibody mogamulizumab recognizes the receptor N-terminus. Structural characterization of the receptor–Fab complex revealed that antibody recognition depends on a highly localized conformational epitope whose integrity is essential for both therapeutic efficacy and clinically observed resistance mutations [21]. However, unlike leronlimab, mogamulizumab does not exert its primary activity through a direct modulation of receptor signaling but through the activation of antibody-dependent cellular cytotoxicity (ADCC), in which receptor recognition serves as a targeting mechanism for immune effector recruitment.

The comparison between CCR5 and CCR4 illustrates a structural dissociation between recognition mode and pharmacological mechanism: both biologics exploit conformational epitopes formed by the receptor N-terminus and extracellular loops, yet their therapeutic effects are mediated by entirely different processes.

### 2.2. Orthosteric Pocket Recognition: Beyond Conventional Ligand Mimicry

The compact extracellular architecture of most Class A GPCRs restricts direct access to the orthosteric binding pocket, limiting recognition by conventional monoclonal antibodies. Nanobodies overcome this structural constraint through elongated CDR3 loops that adopt β-hairpin or β-finger conformations capable of penetrating the extracellular vestibule and directly engaging the orthosteric cavity (Figure 2B). Unlike endogenous ligands, these biologics simultaneously establish deep interactions within the ligand-binding pocket and extensive contacts with the surrounding extracellular architecture, coupling receptor affinity with subtype selectivity and conformational regulation [3,14].

The atypical chemokine receptor ACKR3 provides a well-characterized example of orthosteric recognition by an extracellular-binding nanobody. VUN701 acts as a selective and reversible competitive antagonist of CXCL11- and CXCL12-induced β-arrestin2 recruitment, with nanomolar functional affinity for ACKR3 and no detectable inhibition of the closely related chemokine receptors CXCR3 and CXCR4. The solution NMR structure of VUN701 (PDB ID: 8UEK) revealed an extended CDR3 β-hairpin comprising residues 91–109. Deletion of CDR3 almost completely abolished receptor inhibition, whereas deletion of CDR1 or CDR2 retained substantial antagonistic activity, identifying the extended CDR3 as the principal determinant of orthosteric engagement [14].

Because an experimentally resolved ACKR3-VUN701 complex is not currently available, the receptor–nanobody interface was defined through AlphaFold2 modeling, molecular dynamics simulations, and systematic mutagenesis. The integrative structural model positions the CDR3 β-hairpin deep within the ACKR3 orthosteric pocket. Lys100^CDR3^ is positioned between Asp275^6.58^ and Glu290^7.27^ (superscripts refer to Ballesteros-Weinstein numbering) [22], forming a charge-complementary interaction network, whereas Arg103^CDR3^ is oriented toward Asp179^4.61^ and may establish a salt bridge. At the extracellular surface, His32^CDR1^ is positioned to form a cation–π interaction with Phe285^ECL3^. Alanine substitution identified His32^CDR1^, Asp56^CDR2^, Lys100^CDR3^, Arg103^CDR3^, and Phe106^CDR3^ as major contributors to VUN701 activity, with the K100A substitution reducing functional affinity below the detection limit. Complementary receptor mutagenesis showed that Asp275^6.58^, Phe285^ECL3^, and Glu290^7.27^ are particularly important for nanobody recognition [14].

Beyond defining receptor recognition, the integrative structural model also provides a mechanistic explanation for VUN701 antagonism. Comparison with the CXCL12-bound ACKR3 structure (PDB ID: 7SK5) [23] indicates that agonist binding promotes an approximately 5 Å upward displacement of TM7, enabling formation of the polar network involving Asp90^2.50^, Asn311^7.49^, and Tyr315^7.53^ within the NPxxY activation region. VUN701 positions CDR1 and CDR2 above the extracellular end of TM7 and restricts this displacement, thereby disfavoring stabilization of the active receptor conformation. The functional relevance of this movement was supported by engineering an A271C^6.54^-L293C^7.30^ disulfide bond at the TM6-TM7 interface, which restricted receptor activation without substantially altering CXCL12 potency.

VUN701 selectivity arises from the combined recognition of conserved and receptor-specific structural elements. Interactions of the extended CDR3 with Asp275^6.58^ and Glu290^7.27^ provide high-affinity engagement of the conserved orthosteric pocket, whereas distal contacts involving CDR1, ECL2, ECL3, and the receptor N-terminus discriminate ACKR3 from related chemokine receptors. Phe285^ECL3^ is particularly relevant because the corresponding positions are occupied by Arg288 in CXCR3 and Gln272 in CXCR4. However, introducing Phe at these positions or transferring ACKR3 ECL3 alone was insufficient to confer VUN701 sensitivity, demonstrating that selectivity emerges from the complete composite interface and cannot be attributed to a single residue [14].

Although VUN700, VUN701, and VUN702 recognize highly overlapping receptor epitopes, they display distinct pharmacological profiles ranging from competitive antagonism to inverse agonism [24], indicating that pharmacological activity depends on the conformational state stabilized by the nanobody.

The μ-opioid receptor (μOR) extends this recognition paradigm by demonstrating how orthosteric binding can be integrated with extensive extracellular loop recognition to achieve receptor subtype selectivity. The cryo-EM structure of μOR bound to the antagonist nanobody NbE (PDB ID: 8QOT) [25] revealed an unprecedented binding mode in which the nanobody approaches the receptor entirely from the extracellular side while inserting an elongated CDR3 β-hairpin deeply into the orthosteric binding cavity. Unlike conventional opioid antagonists, NbE establishes a dual interaction network involving both the conserved orthosteric pocket and the structurally divergent extracellular vestibule.

The tip of the CDR3 β-hairpin, formed primarily by Tyr106, Phe107, and Tyr108, penetrates the orthosteric pocket and forms an extensive hydrophobic interaction network with residues lining the ligand-binding cavity. In particular, Tyr108 stacks against Leu219^ECL2^, creating an interaction that is absent from currently available small-molecule antagonists and peptide agonists. Additional hydrogen bonds and salt bridges are established between Lys209^ECL2^, Arg211^ECL2^, and Gln212^ECL2^ of μOR and Glu111, Tyr102, Ser103, and Thr35 of NbE, while Lys303^6.58^ forms a specific hydrogen bond with Ser103 of the nanobody. Together, these interactions stabilize the receptor in an inactive conformation while preventing orthosteric ligand binding.

The structural basis of subtype selectivity resides predominantly within the extracellular loops, with the conserved orthosteric cavity contributing mainly to high-affinity receptor engagement. Sequence comparison among μ-, δ-, and κ-opioid receptors revealed substantial divergence in ECL2 and ECL3, which constitute the principal recognition surfaces for NbE. Alanine substitution of the K209-R211-Q212-L219 (“KRQL”) motif within ECL2 reduced NbE binding by approximately 50%, whereas mutation of Glu310 in ^ECL3^ similarly impaired receptor recognition. Conversely, introducing μOR-specific residues into δOR transformed a non-binding receptor into an efficient NbE binder. In particular, the triple mutant D193Q/M199T/L300W conferred robust nanobody binding, while replacement of the δOR ECL3/TM7 region with the corresponding μOR sequence further increased receptor recognition. These gain-of-function experiments demonstrate that subtype selectivity emerges from the coordinated contribution of orthosteric and extracellular vestibule interactions and cannot be attributed to individual residues alone.

Comparison of the NbE-bound structure with antagonist-bound μOR structures further revealed that nanobody binding induces measurable rearrangements of ECL2, ECL3, TM6, and TM7, highlighting the extracellular vestibule as a dynamic recognition surface and not just a passive entrance to the orthosteric pocket.

The apelin receptor (APJ) provides a striking example of how subtle modifications at the receptor–biologic interface can profoundly alter pharmacological outcome without changing the overall recognition mode. Cryo-EM structures of APJ bound to the antagonist nanobody JN241 (PDB ID: 8XQJ) and its agonist derivative JN241-9 (PDB ID: 8Z7J) [26] revealed nearly identical binding orientations, demonstrating that conversion from antagonism to agonism does not require recognition of a different epitope but instead results from minimal remodeling of the orthosteric interface.

JN241 binds APJ through an elongated CDR3 that penetrates the extracellular vestibule and occupies the orthosteric cavity. The nanobody establishes extensive interactions with the extracellular loops and the upper regions of TM2, TM3, TM6, and TM7 while maintaining the receptor in an inactive conformation. Functional analyses showed that the antagonist prevents productive rearrangement of the conserved activation microswitches despite occupying essentially the same receptor surface as the endogenous peptide ligand.

The agonistic nanobody JN241-9 differs from JN241 by the insertion of a single tyrosine residue within CDR3. Structural comparison demonstrated that this additional residue extends toward Tyr299^7.43^, located near the base of the orthosteric cavity, establishing new hydrophobic contacts that propagate through TM7 toward the conserved NPxxY activation motif. These interactions induce inward displacement of the extracellular end of TM7 and the conformational rearrangements required for productive G protein coupling. Consequently, the nanobody is converted from a competitive antagonist into a full agonist while preserving virtually the same overall receptor-binding geometry.

Mutagenesis confirmed the importance of this interaction network. Substitutions affecting Tyr299^7.43^ markedly reduced JN241-9-mediated receptor activation without significantly altering receptor expression or antagonist binding, demonstrating that agonism arises from specific stabilization of the active-state conformation without requiring increased receptor affinity. These observations establish Tyr299^7.43^ as a critical structural switch linking extracellular nanobody recognition to activation of the transmembrane signaling core.

Minimal modification of a single CDR loop is therefore sufficient to redirect APJ conformational equilibria and convert antagonism into agonism while preserving the overall receptor-binding geometry.

### 2.3. Intracellular Active-State Recognition: Stabilizing the Signaling Interface

Activation of Class A GPCRs is associated with a conserved series of conformational rearrangements, most notably the outward displacement of transmembrane helix 6 (TM6), accompanied by smaller movements of TM5 and TM7. These structural transitions generate a large intracellular cavity that accommodates the C-terminal α5 helix of heterotrimeric G proteins. Intracellular-binding nanobodies recognize this same cavity, selectively stabilizing active receptor conformations and enabling high-resolution structural characterization of GPCR signaling complexes (Figure 2C). Because this intracellular interface is highly conserved across Class A GPCRs, these biologics preferentially recognize conserved conformational states with limited dependence on receptor-specific sequence features, making them valuable molecular tools for investigating GPCR activation.

The β2-adrenergic receptor (β2AR) provided the first structural demonstration of intracellular conformational recognition by a nanobody. In the crystal structure of β2AR bound to the high-affinity agonist BI-167107 and the intracellular-binding nanobody Nb80 (PDB ID: 3P0G) [12], the elongated CDR3 loop of Nb80 inserts deeply into the cytoplasmic cavity formed by TM3, TM5, TM6, and TM7. This binding geometry closely overlaps that subsequently observed for the C-terminal α5 helix of Gα*_s_* in the β2AR-G_s_ complex, establishing Nb80 as the first structural mimic of G protein coupling. The subsequent β2AR-G*_s_* structure confirmed this structural mimicry, showing that the α5 helix of Gα_s_ occupies essentially the same intracellular cavity stabilized by Nb80. This close structural correspondence established that Nb80 reproduces the physiological receptor–transducer interface, validating its use as a faithful surrogate for G protein coupling.

Nb80 captures the receptor in a G protein-like active conformation by stabilizing the conserved microswitch network linking agonist binding to intracellular signaling. Structurally, nanobody binding is associated with an approximately 11 Å outward displacement of the cytoplasmic end of TM6, accompanied by coordinated rearrangements of TM5 and TM7 [12]. At the molecular level, receptor activation disrupts the inactive-state ionic interaction involving Arg131^3.50^ and Glu268^6.30^ and reorganizes the conserved transmission switch formed by Ile121^3.40^, Pro211^5.50^, and Phe282^6.44^. Rotation of Phe282^6.44^ contributes to the outward movement of TM6, while rearrangement of the NPxxY motif repositions Tyr326^7.53^ into its active-state orientation. Higher-resolution β2AR structures further revealed a water-mediated hydrogen bond between Tyr219^5.58^ and Tyr326^7.53^, linking TM5 and TM7 and reinforcing stabilization of the intracellular active-state architecture.

Biochemical characterization showed that Nb80 preferentially binds agonist-occupied β2AR and increases agonist affinity, reproducing the positive allosteric effect normally exerted by G_s_. Thus, Nb80 primarily selects and stabilizes a pre-existing active conformational ensemble. The original structural study did not use systematic receptor interface mutagenesis to assign individual Nb80 contacts; therefore, the contribution of the nanobody is most rigorously described through its binding geometry, conformational selectivity, and stabilization of the conserved active-state microswitch network.

The subsequent β2AR-G*_s_* structure (PDB ID: 3SN6) [12] confirmed the physiological relevance of the Nb80-stabilized state, showing that the α5 helix of Gα*_s_* occupies the same intracellular cavity while receptor coupling produces a larger, approximately 14 Å outward movement of TM6 and an intracellular extension of TM5. In this complex, Nb35 does not directly recognize β2AR but binds across the Gα*_s_*-Gβ interface, stabilizing the nucleotide-free receptor–G*_s_* complex during structural determination.

μOR demonstrates that the same intracellular cavity can be recognized through a distinct binding geometry. In the 2.1 Å crystal structure of μOR bound to the high-affinity agonist BU72 and Nb39 (PDB ID: 5C1M), the nanobody recognizes the intracellular surface formed by ICL2, ICL3, helix 8, and the cytoplasmic ends of the transmembrane helices. Unlike Nb80, Nb39 penetrates less deeply into the intracellular cavity and relies extensively on hydrogen bonds involving framework residues in addition to CDR-mediated interactions, demonstrating that distinct nanobody architectures can converge on stabilization of the same active receptor conformation [27,28].

Nb39 binding stabilizes the active μOR architecture, including the outward displacement of TM6 and rearrangement of the conserved E/DRY and NPxxY motifs. In the active receptor, Asn^7.49^ and Tyr^7.53^ interact with Tyr^5.58^ and the backbone carbonyl of Leu^3.43^ through a water-mediated polar network connecting TM3, TM5, and TM7. This network provides a structural pathway linking agonist recognition to organization of the intracellular signaling interface.

The κ-opioid receptor (κOR) further illustrates that conformational nanobodies recognize conserved active-state architectures across related GPCRs. The crystal structure of κOR bound to the agonist MP1104 and the active-state nanobody Nb39 (PDB ID: 6B73) [29], revealed that Nb39 occupies the intracellular transducer-binding cavity through a binding mode closely resembling that observed for μOR, stabilizing the outward displacement of TM6 and the active organization of the conserved intracellular microswitch network. Despite differences in the orthosteric binding pocket and extracellular regions, the intracellular signaling interface remains sufficiently conserved to support recognition by the same nanobody [30].

Nb39 was subsequently repurposed as a conformational biosensor in living cells. Fluorescently labelled Nb39 selectively translocated from the cytoplasm to activated κOR upon agonist stimulation, allowing direct visualization of receptor activation with high spatial and temporal resolution. These experiments extended the application of intracellular-binding nanobodies from structural stabilization to dynamic monitoring of GPCR activation in living cells.

Although intracellular nanobodies can selectively stabilize active receptor conformations, their ability to distinguish signaling-specific substates requires careful interpretation. Nanobodies such as Nb80 and Nb39 primarily recognize intracellular architectures associated with G protein coupling and have been extensively used as conformational stabilizers and biosensors. Their structural resemblance to G protein-bound states does not, however, establish that nanobody binding selectively promotes G protein signaling over β-arrestin recruitment. In principle, engineered intracellular binders could discriminate receptor conformations associated with distinct transducer preferences, provided that such selectivity is supported by comparative structural and functional evidence. Thus, current structural applications primarily demonstrate active-state recognition, while the selective stabilization of signaling-biased conformations represents an important direction for future biologic engineering.

### 2.4. Allosteric Recognition: Modulating Receptor Function Beyond the Orthosteric Site

Allosteric recognition represents a distinct mechanism of GPCR modulation in which biologics engage receptor surfaces outside the orthosteric pocket and influence receptor activity through conformational coupling with the transmembrane core. Extracellular allosteric surfaces can provide structurally variable recognition platforms, creating opportunities for receptor subtype selectivity while preserving orthosteric ligand binding (Figure 2D).

The α1A-adrenergic receptor (α1AAR) provides a structural example of extracellular conformational recognition. Cryo-EM structures of α1AAR showed that the extracellular nanobody Nb29 binds the extracellular vestibule of the receptor while allowing simultaneous occupancy of the orthosteric pocket by the selective agonist oxymetazoline. Nb29 preferentially recognizes the oxymetazoline-bound receptor compared with apo, noradrenaline-bound, and antagonist-bound states, demonstrating conformational selectivity for a specific agonist-stabilized receptor state [31].

The cryo-EM structure of the oxymetazoline–α1AAR-Nb29 complex revealed that nanobody binding stabilizes the extracellular region of the receptor. Comparison of active and inactive α1AAR structures further showed conformational rearrangements involving ECL2 and the extracellular regions of TM4 and TM7, illustrating the structural plasticity of the extracellular vestibule during receptor activation.

Functional characterization supported the conformational selectivity of Nb29. The nanobody preferentially bound oxymetazoline-occupied α1AAR and shifted agonist competition curves, with a stronger effect on α1AAR than on the α1B- and α1D-adrenergic receptor subtypes. These findings support a coupling between extracellular nanobody recognition and the conformational state of the receptor.

A complementary mechanism is observed in rhodopsin. The cryo-EM structure of rhodopsin bound to the extracellular nanobody Nb2 (PDB ID: 8FD0) [32] revealed that the nanobody recognizes a conformational epitope formed by the receptor N-terminus, ECL2, and native N-linked glycans, without contacting the retinal-binding pocket and stabilizing the extracellular architecture characteristic of the inactive receptor. Structural comparison with inactive and Meta II conformations demonstrated that nanobody binding restricts the extracellular rearrangements required for retinal isomerization and receptor activation. Consequently, receptor inhibition results from stabilization of the inactive conformational ensemble without direct competition with the endogenous ligand.

Nucleic acid aptamers extend the concept of extracellular allosteric recognition beyond protein-based biologics. RNA aptamers directed against the β2-adrenergic receptor were isolated by SELEX using receptors stabilized in distinct conformational states, allowing enrichment of aptamers that preferentially recognize inactive or agonist-induced receptor conformations [33].

Although experimentally resolved GPCR-aptamer structures are not yet available, biochemical and biophysical studies showed that these aptamers discriminate between β2AR conformational states without directly competing with orthosteric ligand binding. Their conformational selectivity and allosteric effects therefore support an extracellular mode of receptor modulation, although the molecular architecture of the receptor–aptamer interface remains unresolved.

### 2.5. Structural Design Principles Emerging from Class A GPCRs

Comparison of structurally resolved Class A GPCR–biologic complexes reveals that the diversity of biologic recognition ultimately arises from a limited number of structural principles imposed by the conserved seven-transmembrane receptor architecture (Figure 2).

The first principle concerns the extent and structural organization of the receptor surface accessible to biologics. Because most Class A GPCRs lack large extracellular domains, biologic recognition is generally confined to composite surfaces formed by the N-terminus, extracellular loops, and extracellular ends of the transmembrane helices. These discontinuous surfaces provide sufficient structural diversity to discriminate closely related receptor subtypes despite the high conservation of the transmembrane core. Chemokine receptors represent a notable exception, as their extended N-termini enlarge the accessible extracellular surface and create protein–protein interaction interfaces naturally suited for biologic recognition.

A second principle emerges from comparison of orthosteric biologics. The ACKR3, μOR, and APJ examples show that different regions of the receptor–biologic interface can make distinct contributions to affinity and selectivity. Deep engagement of the orthosteric cavity provides extensive receptor contacts, whereas interactions with structurally variable extracellular regions can encode subtype discrimination. Orthosteric recognition by biologics therefore combines engagement of a relatively conserved ligand-binding cavity with recognition of more divergent extracellular surfaces.

A third principle concerns the relationship between recognition geometry and pharmacological outcome. Epitope location alone does not determine biologic function: biologics engaging highly overlapping receptor surfaces can stabilize different conformational states and consequently produce antagonism, inverse agonism, agonism, or allosteric modulation. The APJ-directed nanobodies provide a particularly clear example, as insertion of a single tyrosine within CDR3 converts an antagonist into a full agonist while largely preserving the overall receptor-binding mode. Pharmacological activity is therefore determined by how the interaction reshapes the receptor conformational equilibrium in addition to where binding occurs.

Intracellular recognition reveals a complementary architectural principle. Activation of the conserved 7TM core generates a transducer-binding cavity with a shared three-dimensional organization across Class A GPCRs. Conformational nanobodies such as Nb80 and Nb39 can therefore recognize related active-state architectures across receptors with divergent extracellular surfaces. Their binding stabilizes conformations characterized by outward displacement of TM6 and coordinated reorganization of the E/DRY, PIF, and NPxxY microswitches, explaining their utility as structural chaperones and conformational biosensors.

Extracellular conformational recognition further demonstrates that receptor modulation can occur without direct competition for the orthosteric site. Structural studies of extracellular nanobodies, together with biochemical evidence from conformationally selective aptamers, show that extracellular receptor states can be selectively recognized and coupled to changes in receptor function. These observations extend the accessible recognition landscape of Class A GPCRs beyond the orthosteric pocket while highlighting extracellular conformational plasticity as an additional determinant of biologic engagement.

Collectively, Class A GPCR structures identify two interdependent determinants of biologic recognition: the accessibility and three-dimensional organization of receptor surfaces, which define where a biologic can engage the receptor, and the conformational state stabilized by that interaction, which shape its pharmacological consequences. The compact extracellular architecture of most Class A receptors restricts conventional biologics to exposed composite epitopes, whereas nanobodies can additionally access orthosteric and intracellular cavities. Across these distinct binding modes, biologic function ultimately depends on selective stabilization of functionally relevant receptor conformations. These principles provide a structural basis for engineering Class A GPCR-targeted biologics with improved subtype selectivity and controlled pharmacological activity.

## 3. Structural Determinants of Biologic Recognition in Class B GPCRs

Class B GPCRs display a recognition landscape that differs fundamentally from that of Class A receptors because endogenous peptide binding is governed by a large N-terminal extracellular domain (ECD). This domain creates an extensive interaction surface that can be exploited by antibodies, engineered peptides, and, more recently, bitopic biologics [34,35,36].

Structural studies reveal three recurrent modes of biologic recognition in Class B GPCRs [37,38]. Antibodies predominantly engage conformational epitopes within the ECD, preventing peptide binding or restricting extracellular rearrangements associated with receptor activation. Engineered peptide agonists preserve the physiological two-domain binding mechanism while incorporating modifications that improve stability and pharmacological properties. More recently, bitopic biologics have combined extracellular receptor recognition with transmembrane receptor activation, simultaneously exploiting both structural modules within a single molecular construct.

### 3.1. Recognition of Extracellular Domains: The Dominant Structural Determinant in Class B GPCRs

The large extracellular domain (ECD) represents the defining structural feature of Class B GPCRs and provides an extensive solvent-exposed platform for biologic recognition. Because the ECD participates directly in endogenous peptide binding and communicates structurally with the transmembrane core, biologics can influence receptor function by interfering with peptide recognition or by modifying extracellular conformational states associated with receptor activation.

The calcitonin gene-related peptide receptor (CGRPR) provides one of the most informative examples of extracellular biologic recognition in Class B GPCRs. The functional receptor consists of the calcitonin receptor-like receptor (CLR) associated with receptor activity-modifying protein 1 (RAMP1), which together generate a composite extracellular interface responsible for high-affinity CGRP recognition. This quaternary organization creates an extended receptor surface that can be selectively targeted by biologics. The crystal structure of the anti-CGRPR monoclonal antibody erenumab bound to the CLR-RAMP1 extracellular complex (PDB ID: 6UMG) revealed an extensive binding interface involving all six complementarity-determining regions (CDRs) of the antibody heavy and light chains [16,39]. The interaction buries approximately 1340 Å^2^ of receptor-accessible surface, with nearly three quarters of the interface contributed by CLR and the remainder by RAMP1. The heavy chain dominates receptor recognition, whereas the unusually long 21-residue CDR-H3 contributes more than half of the total paratope surface.

The central recognition element is formed by four aromatic residues within CDR-H3 (Tyr103, Tyr104, Tyr109, and Tyr110), which establish an extensive network of hydrophobic and hydrogen bond interactions with both receptor components. In particular, Tyr103 and Tyr104 pack against Trp74 of RAMP1, whereas Tyr109 and Tyr110 interact with Trp72 of CLR and Trp84 of RAMP1, generating an aromatic interaction cluster that constitutes the structural core of the interface. Additional stabilization is provided by Asp54 of CDR-H2, which forms hydrogen bonds with Ser117 and Arg119 of CLR. Alanine substitution of any of the four CDR-H3 tyrosines abolished detectable receptor binding, whereas the D54A mutation accelerated antibody dissociation by approximately fivefold, experimentally confirming the central contribution of these residues to high-affinity recognition.

The receptor epitope comprises 18 CLR residues and five RAMP1 residues. Several CLR-specific positions, including Glu23, Leu24, Glu25, Glu26, Ile41, and His132, contribute directly to antibody recognition, whereas Trp74, Ala70, Asp71, and Trp84 of RAMP1 provide an additional layer of subtype selectivity through their limited conservation among RAMP paralogues. Structural comparison with peptide-bound receptor complexes further demonstrated that the erenumab footprint overlaps the endogenous CGRP-binding surface while inducing an approximately 3 Å displacement of RAMP1 relative to CLR. The overlap between the erenumab footprint and the endogenous CGRP-binding surface therefore provides a structural basis for direct inhibition of peptide docking, while simultaneous engagement of CLR and RAMP1 contributes to receptor complex selectivity.

The glucagon receptor (GCGR) illustrates a complementary strategy for exploiting the Class B extracellular domain. Unlike erenumab, which primarily blocks peptide docking, GCGR-directed antibodies demonstrate that extracellular biologics can also regulate receptor activation by targeting the dynamic structural coupling between the ECD, the stalk region, and the extracellular loops.

The crystal structure of the isolated GCGR extracellular domain bound to the inhibitory monoclonal antibody mAb1 (PDB ID: 4ERS) [40] showed that the antibody occupies the glucagon-binding cleft, directly preventing hormone recognition through steric occlusion. Subsequent structural analysis of the full-length inactive receptor in complex with the negative allosteric modulator NNC0640 and mAb1 (PDB ID: 5XEZ) [41] further demonstrated that antibody recognition extends across the extracellular domain while preserving the overall inactive receptor architecture.

Hydrogen-deuterium exchange experiments identified increased protection of the α helix (Leu32-Leu38) and the β4-L5 region (Lys98-Gln105) following mAb1 binding, identifying these regions as the principal antibody interaction surface. In contrast, the stalk segment (Ile128-Met137) was selectively protected by the functionally distinct antibody mAb23, indicating that the two antibodies recognize different structural determinants within the extracellular receptor architecture [42].

The full-length structure further revealed that the receptor stalk adopts a β-strand conformation closely associated with the ECL1 β-hairpin, forming an integrated extracellular assembly that dynamically couples the ECD with the transmembrane bundle. Molecular dynamics simulations identified extensive hydrophobic interactions between Leu32, Phe33, Trp36, Tyr65, Tyr84, Leu85, Pro86, and Trp87 of the ECD and Tyr202, Lys205, and Ile206 of ECL1. Additional electrostatic interactions involving Glu127, Glu129, Asp208, Asp209, Arg111, and Arg116 reinforce this extracellular network. Disulfide cross-linking between Val130-Leu210 and Gln113-Asp209 significantly altered glucagon binding and receptor activation, experimentally demonstrating the functional importance of ECD-stalk-ECL1 communication.

### 3.2. Recognition Through the Two-Domain Binding Mechanism

The two-domain binding mechanism represents the defining structural principle of peptide recognition in Class B GPCRs. Unlike Class A receptors, ligand recognition is distributed across two structurally and functionally distinct receptor modules that perform complementary roles during receptor activation. The C-terminal portion of the peptide first binds the extracellular domain (ECD), providing high-affinity receptor recognition and orienting the ligand above the transmembrane bundle [38,43]. The peptide N-terminus subsequently inserts into the transmembrane domain (TMD), where it establishes the interactions required for receptor activation and G protein coupling. This structural separation between receptor recognition and receptor activation provides an ideal framework for biologic engineering, allowing optimization of peptide stability, affinity, efficacy, and signaling duration while preserving the endogenous activation mechanism.

The glucagon-like peptide-1 receptor (GLP-1R) provides one of the clearest examples of therapeutic optimization within the constraints of the physiological two-domain binding mechanism. The cryo-EM structure of semaglutide bound to GLP-1R-G_s_ (PDB ID: 7KI0) [44] revealed that the engineered peptide adopts a continuous α-helical conformation virtually indistinguishable from that of endogenous GLP-1, demonstrating that therapeutic optimization preserves the native recognition strategy.

The C-terminal region of semaglutide anchors the peptide to the receptor ECD, whereas the N-terminal residues His7, Aib8, Glu9, and Thr11 penetrate the transmembrane cavity to establish the principal activation interface. Substitution of Ala8 by the conformationally restricted residue Aib8 selectively remodels receptor interactions within TM7. In particular, Aib8 establishes additional contacts with Leu384^7.39^ and Glu387^7.42^, whereas native GLP-1 primarily interacts with Glu387^7.42^ and Leu388^7.43^ (superscripts refer to Wootten numbering) [45]. Functional mutagenesis demonstrated that substitution of Leu384^7.39^ selectively impaired semaglutide-mediated signaling while exerting a substantially smaller effect on endogenous GLP-1, experimentally confirming that limited modifications of individual receptor contacts can substantially alter peptide pharmacology without disrupting the overall binding mechanism.

Semaglutide further incorporates a Lys26-linked C18 fatty diacid, attached through a γ-glutamyl-2xOEG spacer. Structural analysis demonstrated that this lipid modification projects away from the receptor-binding interface and remains highly flexible, explaining how albumin binding and prolonged plasma half-life are achieved without perturbing receptor recognition.

Three-dimensional variability analysis further revealed that the semaglutide-bound complex samples showed pronounced motions involving the ECD, ECL1, ECL3, and the extracellular regions of TM2, TM6, and TM7. Compared with the endogenous GLP-1 complex, semaglutide stabilizes a broader conformational ensemble while preserving the same overall receptor architecture. These observations indicate that therapeutic peptide engineering can modulate receptor dynamics independently of the conserved two-domain binding mechanism.

The parathyroid hormone receptor type 1 (PTH1R) provides a complementary example of how the two-domain architecture can be exploited to prolong receptor signaling. The cryo-EM structure of PTH1R bound to the long-acting agonist LA-PTH and G_s_ (PDB ID: 6NBF) [46] revealed an extended peptide helix simultaneously engaging both receptor domains.

The C-terminal portion of LA-PTH binds the central groove of the extracellular domain, providing high-affinity receptor recognition, whereas the N-terminal residues penetrate deeply into the transmembrane bundle to establish the activation interface. Structural and functional analyses consistently identify residues 1–14 as the principal TMD-engaging pharmacophore, whereas the C-terminal peptide region contributes predominantly to receptor affinity. This organization assigns high-affinity ECD engagement and transmembrane activation to distinct regions of the same peptide.

The first four residues of LA-PTH establish an extensive interaction network within the transmembrane cavity and are indispensable for receptor activation. Consistent with this structural arrangement, the truncated analog LA-PTH(5–36) retains receptor binding but behaves as a competitive antagonist because it lacks the N-terminal activation sequence. Thus, peptide affinity and signaling efficacy can be experimentally uncoupled while preserving the overall receptor-binding architecture.

The full-length structure further demonstrated that insertion of the peptide N-terminus induces partial unwinding of TM6 and formation of the intracellular cavity required for G protein coupling. In contrast, the extracellular domain remained highly dynamic and was resolved in multiple conformational states, indicating that peptide anchoring at the ECD is compatible with substantial extracellular flexibility. Preferential stabilization of the R^0^ state therefore links the engineered receptor-binding properties of LA-PTH with prolonged receptor residence time and sustained cAMP signaling after internalization.

### 3.3. Bitopic Biologics: Integrating Extracellular Recognition with Receptor Activation

Bitopic biologics represent a natural extension of the structural organization that defines Class B GPCRs. The structural separation between extracellular ligand recognition and transmembrane receptor activation creates two distinct yet structurally coupled receptor modules that can be simultaneously exploited within a single biologic [46,47].

The parathyroid hormone receptor type 1 (PTH1R) provides the first experimental demonstration of this design strategy. Guided by the structural organization of active PTH1R-G_s_ complexes, receptor-specific nanobodies recognizing the extracellular domain were covalently linked to truncated PTH agonists, generating biologics that simultaneously engage both structural modules of the receptor [47].

The extracellular nanobody anchors the biologic to the receptor surface and constrains the spatial orientation of the peptide agonist relative to the transmembrane bundle. This structural preorganization facilitates productive insertion of the peptide N-terminus into the activation cavity while maintaining high-affinity recognition of the extracellular domain.

Systematic variation in linker length demonstrated that biological activity depends critically on the geometric relationship between the extracellular recognition module and the transmembrane activation module. Only constructs preserving an appropriate spatial relationship between the extracellular recognition and transmembrane activation modules retained maximal agonist activity, providing direct experimental evidence that receptor–biologic geometry is a determinant of functional activity [47].

Functional characterization further revealed marked improvements in receptor potency, subtype selectivity, and signaling efficacy relative to peptide agonists alone. Nanobody-mediated receptor anchoring also prolonged receptor occupancy, enhanced G_s_-dependent signaling, and reduced β-arrestin recruitment [48].

These findings identify the spatial relationship between extracellular recognition and transmembrane activation modules as a tunable determinant of bitopic biologic activity.

The differential enhancement of Gs-dependent signaling and reduction in β-arrestin recruitment observed for PTH1R-targeted nanobody-peptide conjugates further suggests that biologic architecture can influence transducer preference. However, these functional differences do not by themselves establish selective stabilization of structurally distinct G protein- or β-arrestin-preferring receptor conformations. Additional structural characterization, together with pathway-resolved functional analyses, is needed to determine whether such bitopic constructs produce genuine signaling bias through conformational selection or primarily alter the kinetics and efficacy of receptor activation [47,48].

### 3.4. Structural Design Principles Emerging from Class B GPCRs

**Figure 3:**
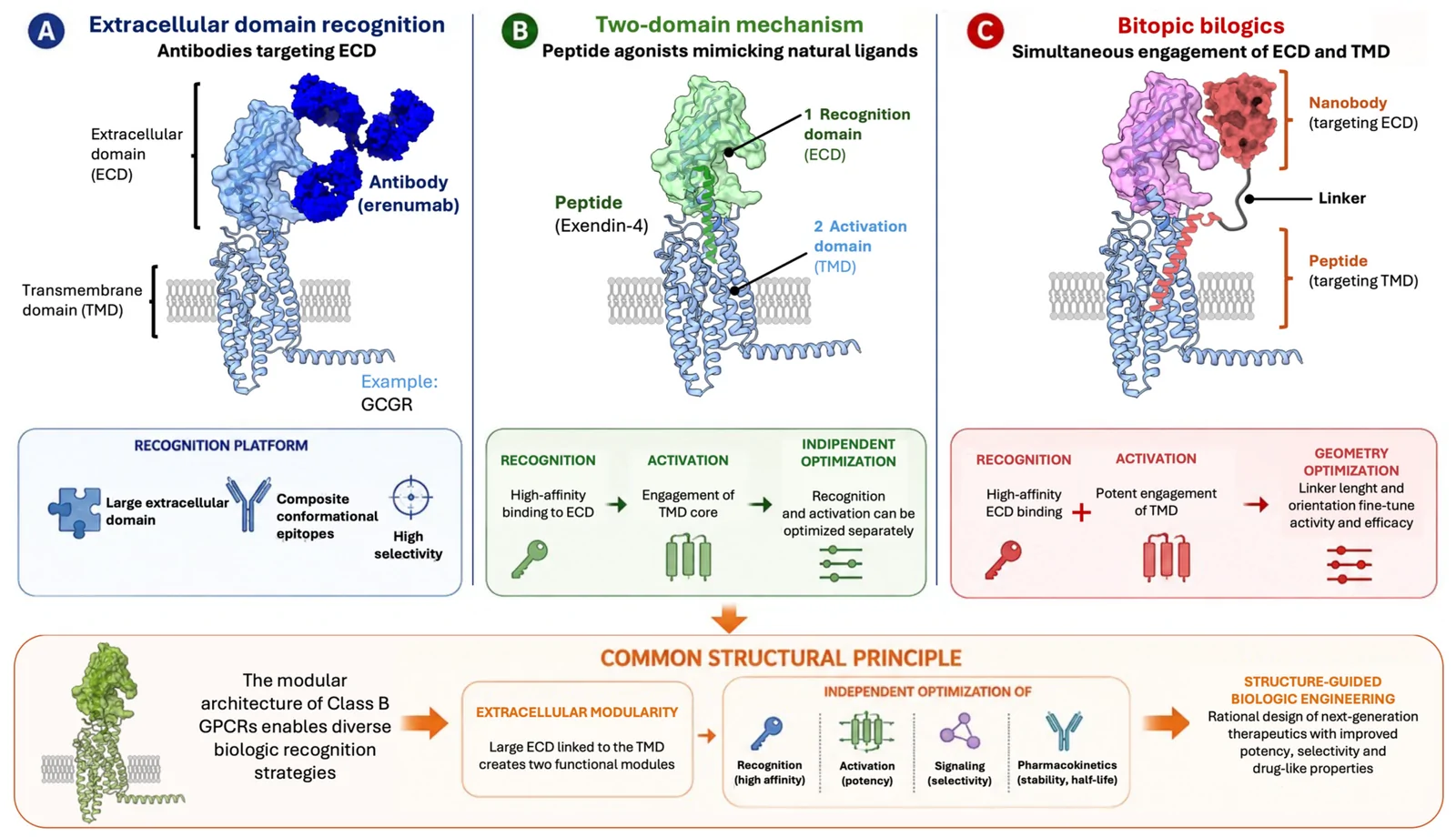
Principal structural paradigms governing biologic recognition in Class B GPCRs. The characteristic extracellular architecture of Class B GPCRs enables three major biologic recognition strategies. (**A**) Therapeutic antibodies recognize composite conformational epitopes within the extracellular domain (ECD), preventing peptide binding or restricting extracellular domain mobility to stabilize inactive receptor conformations. (**B**) Engineered peptide agonists exploit the physiological two-domain binding mechanism, in which the ECD mediates high-affinity ligand recognition while the transmembrane domain (TMD) governs receptor activation. (**C**) Bitopic biologics simultaneously engage extracellular and transmembrane receptor modules, integrating receptor recognition with activation to enhance potency, selectivity, and signaling efficacy. The receptor shown in all panels corresponds to the AlphaFold-predicted structure of the human glucagon receptor (GCGR), used as a representative Class B GPCR for illustrative purposes. The immunoglobulin displayed as a blue surface in (**A**) was derived from PDB ID: 1IGT. The peptide shown as a green cartoon in (**B**) corresponds to Exendin-4 and was derived from PDB ID: 6GDZ. The nanobody depicted in (**C**) was derived from PDB ID: 7MFV.

Comparison of structurally resolved Class B GPCR–biologic complexes reveals how expansion of the extracellular receptor architecture creates recognition opportunities that are largely absent from the more compact extracellular surface of most Class A GPCRs (Figure 3).

The first defining principle is architectural accessibility. The large Class B ECD provides an extended solvent-accessible surface capable of supporting high-affinity recognition by conventional antibodies and other large biologics. Importantly, this surface is functionally integrated with endogenous peptide recognition and with the transmembrane core. Biologic engagement of the ECD can therefore influence both ligand occupancy and receptor conformational dynamics [16,40,42].

A second principle is functional modularity. The physiological two-domain binding mechanism spatially separates high-affinity extracellular recognition from transmembrane activation. This organization allows individual components of a biologic to be modified without abolishing the fundamental receptor activation mechanism, as illustrated by engineered peptide agonists and nanobody-peptide conjugates [19,46,49].

A third principle concerns interaction geometry. Bitopic biologics demonstrate that pharmacological activity depends not only on engagement of the appropriate receptor modules but also on their spatial relationship within the receptor–biologic complex. Linker architecture and molecular orientation can therefore influence potency, signaling efficacy, and transducer preference even when the targeted receptor regions remain unchanged.

Collectively, Class B GPCR structures reinforce the two determinants identified for Class A receptors while extending them to a substantially larger extracellular architecture. The extent and organization of the accessible receptor surface define the available modes of biologic engagement, whereas conformational coupling between the ECD and the 7TM core determines how extracellular binding is translated into receptor activation and signaling. The modular architecture of Class B GPCRs additionally creates an opportunity to engineer recognition and activation as partially separable components of the same biologic.

## 4. Structural Determinants of Biologic Recognition in Class C and Class F GPCRs

Class C and Class F GPCRs constitute the structured extracellular branch of the GPCR superfamily. Despite recognizing distinct endogenous ligands and performing different physiological functions, both receptor families rely on large extracellular regions that actively participate in ligand recognition, receptor activation, receptor dimerization, and allosteric regulation [17].

The available structures reveal two broad recognition strategies: conformational recognition of the Venus Flytrap (VFT) architecture in Class C receptors, where biologic binding can also influence intersubunit communication, and recognition of cysteine-rich domains (CRDs) in Class F receptors, where biologics can regulate ligand engagement and receptor assembly.

### 4.1. Recognition of Dynamic Venus Flytrap Conformations

The Venus Flytrap (VFT) domain constitutes a major recognition platform for biologics targeting Class C GPCRs. In contrast to the extracellular domains of Class B receptors, the VFT undergoes large-scale rearrangements among inactive, intermediate, and active states during receptor activation. This conformational plasticity creates structurally distinct extracellular states that can be selectively recognized and stabilized by biologics [17,50].

The metabotropic glutamate receptor 5 (mGlu5) provides the first structural demonstration that biologics can selectively stabilize intermediate conformational states of the Venus Flytrap domain. Crystal and cryo-EM structures of mGlu5 in complex with the positive allosteric nanobody Nb43 (PDB IDs: 6N50, 6N51, 6N52) [17] revealed that Nb43 binds the apex of the VFT through predominantly polar interactions involving two receptor loops encompassing residues 345–357 and 365–375. Recognition is mediated predominantly by CDR2 and CDR3, whose architecture positions the nanobody over a shallow groove located at the apex of the VFT.

Importantly, Nb43 does not directly induce closure of the ligand-binding cleft. Instead, structural comparison demonstrated that the nanobody stabilizes an active open-open (Aoo) extracellular conformation characterized by close apposition of the VFT lower lobes despite the ligand-binding domains remaining open. Agonist binding subsequently converts this intermediate into the fully active Acc state through closure of the VFT and formation of the cysteine-rich domain (CRD) interface. These observations demonstrate that VFT closure is sufficient but not strictly required for productive receptor activation.

Biochemical characterization further demonstrated positive cooperativity between Nb43 and orthosteric agonists. Nb43 increased agonist affinity and potentiated receptor signaling while exhibiting little intrinsic activity in the absence of agonist. Molecular dynamics simulations further indicated that Nb43 restricts spontaneous opening of the extracellular domain without locking the receptor into a fully closed state, thereby stabilizing a signaling-competent intermediate along the activation pathway.

The calcium-sensing receptor (CaSR) provides a complementary example in which biologic recognition is strongly dependent on the conformational state of the extracellular domain. Cryo-EM structures of CaSR in complex with the inhibitory nanobody NB2D11 (PDB ID: 7E6U) [51] demonstrated that the nanobody binds the lower lobe of the VFT and stabilizes the inactive receptor conformation by preventing formation of the active dimer interface. Functional analyses showed that receptor inhibition results from restriction of the extracellular conformational rearrangements required for productive activation without direct competition with calcium-binding sites [52,53].

A complementary mechanism is observed for the positive allosteric nanobody Nb4, which selectively recognizes the active extracellular conformation formed in the presence of the endogenous co-agonists Ca^2+^ and L-tryptophan. Nb4 therefore potentiates receptor signaling by stabilizing an activation-competent VFT conformation while preserving the physiological mechanism of ligand recognition. NB2D11 and Nb4 therefore illustrate how biologics targeting the same extracellular domain can stabilize opposite conformational states and produce negative or positive allosteric modulation.

Because Class C receptor activation requires coordinated rearrangements of the two protomers, stabilization of a VFT conformation can also influence intersubunit communication. Biologic recognition of the extracellular domain can therefore propagate beyond the directly engaged protomer and modify the cooperative conformational transitions of the receptor dimer.

### 4.2. Recognition of Frizzled Cysteine-Rich Domains

Class F GPCRs possess an extracellular architecture distinct from both the Venus Flytrap domains of Class C receptors and the peptide-binding extracellular domains of Class B GPCRs. Frizzled receptors contain an N-terminal cysteine-rich domain (CRD) that directly recognizes Wnt ligands through a conserved hydrophobic groove accommodating the Wnt palmitoleate moiety. This lipid-binding surface therefore represents the principal extracellular recognition platform available to biologics. Depending on the location and geometry of the interaction, biologics can block Wnt engagement, interfere with ligand-induced receptor assembly, or be incorporated into synthetic agonists that promote productive Frizzled-co-receptor clustering [54,55].

Structural studies of monoclonal antibodies directed against several Frizzled receptors established that the Wnt lipid-binding groove constitutes a recurrent inhibitory epitope. Crystal structures were determined for the F2.I Fab-FZD5 CRD complex (PDB ID: 6O39) and for the F7.B Fab-FZD7 CRD and F6 Fab-FZD7 CRD complexes (PDB IDs: 6O3A and 6O3B, respectively) [54]. All three antibodies recognize a common surface on the CRD that overlaps the binding site for the palmitoleic acid modification of Wnt proteins. Antibody binding therefore prevents productive insertion of the lipid moiety into the CRD groove and inhibits the earliest extracellular step of Wnt-Frizzled recognition.

Despite targeting a conserved functional site, the antibodies display different patterns of Frizzled cross-reactivity. Structural comparison showed that broad or restricted receptor recognition depends on how each paratope accommodates sequence variation surrounding the conserved lipid-binding pocket. Structure-guided modification of the antibody interfaces was consequently used to alter receptor specificity, pharmacokinetic exposure, and tolerability while retaining blockade of Wnt signalling. Variation in contacts with residues surrounding the conserved groove therefore provides a structural basis for the different cross-reactivity profiles observed among these antibodies.

The functional consequences of this recognition mode extend beyond simple ligand competition. Wnt-associated lipids contribute to CRD engagement and can participate in receptor dimerization or higher-order assembly. Antibodies occupying this region may therefore simultaneously impair Wnt binding and prevent formation of productive extracellular receptor complexes. The CRD lipid-binding groove thus functions as both a ligand-recognition site and an organisational surface controlling receptor assembly.

The FZD3-directed nanobody Nb8 provides a complementary example in which recognition of a less conserved CRD surface produces high receptor-subtype selectivity. The 1.8 Å crystal structure of the FZD3 CRD-Nb8 complex (PDB ID: 8Q7O) revealed an extensive interface at the base of the lipid-binding groove. The receptor and nanobody bury approximately 1300 Å^2^ and 1360 Å^2^, respectively, and the interface is dominated by hydrophobic interactions involving all three CDRs and adjacent framework regions [54].

At the centre of the interface, Met116 of FZD3 establishes hydrophobic contacts with Tyr37, Asn96, and Leu98 of Nb8. In addition, Lys112 of FZD3 forms hydrogen bonds with Glu103 and Asp105 of the nanobody. These residues contribute directly to subtype discrimination: Met116 is unique to FZD3 among human Frizzled CRDs, whereas Lys112 is largely restricted to the FZD3/FZD6 subgroup. The limited conservation of the complete epitope explains the strong preference of Nb8 for FZD3 compared with other Frizzled family members.

Nb8 wraps around the CRD through a concave surface formed by its CDRs and binds immediately adjacent to the Wnt lipid-binding groove. Structural superposition with a Wnt-bound Frizzled complex indicated that Thr52 of Nb8 would sterically obstruct insertion of the Wnt palmitoleate moiety. Additional contacts involving Tyr37, Leu47, Thr52, and Asn96 stabilise a narrowed groove conformation in which the end of CRD helix 3 is displaced by approximately 5.9 Å relative to a lipid-accommodating Frizzled structure. Biolayer interferometry confirmed that Nb8 competes with Wnt5a for FZD3 CRD binding, supporting a mechanism involving both direct steric occlusion and conformational restriction of the lipid-binding groove.

Structural superposition also suggested that Nb8 would interfere with formation of the Wnt-induced CRD dimer interface. The nanobody may therefore inhibit both ligand engagement and receptor assembly, connecting recognition of a subtype-specific CRD epitope with disruption of the higher-order extracellular organisation required for signalling [56].

The same recognition module was subsequently converted into a synthetic agonist by fusing Nb8 to the C-terminal domain of Dickkopf-1, which recruits the LRP5/6 co-receptor. The resulting Nb8-DKK1C Wnt surrogate induced β-catenin-dependent signalling selectively in FZD3-expressing cells. Thus, the pharmacological output of CRD recognition is determined by the molecular context in which the targeting module is presented: Nb8 alone competes with endogenous Wnt, whereas linkage to a co-receptor-binding module promotes productive receptor clustering and agonism.

The antibody and nanobody complexes reveal two complementary features of CRD recognition. The conserved Wnt lipid-binding groove provides a functional surface for broad inhibition of ligand engagement, whereas less conserved surrounding regions can support subtype-selective recognition. The Nb8-DKK1C construct further shows that the pharmacological consequence of CRD targeting depends on the architecture of the biologic itself, as the same FZD3-recognition module can support antagonism or engineered agonism depending on whether co-receptor recruitment is incorporated into the molecular design.

Although extracellular recognition of Frizzled receptors by biologics has been predominantly characterized at the cysteine-rich domain (CRD), additional receptor regions can support biologic engagement. Notably, the cryo-EM structure of FZD3 bound to the intracellular nanobody Nb9 (PDB ID: 8QW4) [55] demonstrates that the cytoplasmic surface of the seven-transmembrane core can accommodate biologic binding, with Nb9 interfering with Dishevelled recruitment and G protein coupling. In contrast, experimentally resolved Frizzled–biologic complexes directly engaging extracellular loops or the extracellular-facing surface of the transmembrane core have not been identified in the structural evidence considered here. Although these regions may represent potential recognition sites, their accessibility to larger biologic modalities and their associated conformational consequences remain to be established. Thus, current structural evidence supports both extracellular CRD-directed and intracellular transmembrane-associated recognition, while direct targeting of extracellular loops remains an opportunity for future investigation.

### 4.3. Structural Design Principles Emerging from Class C and F GPCRs

**Figure 4:**
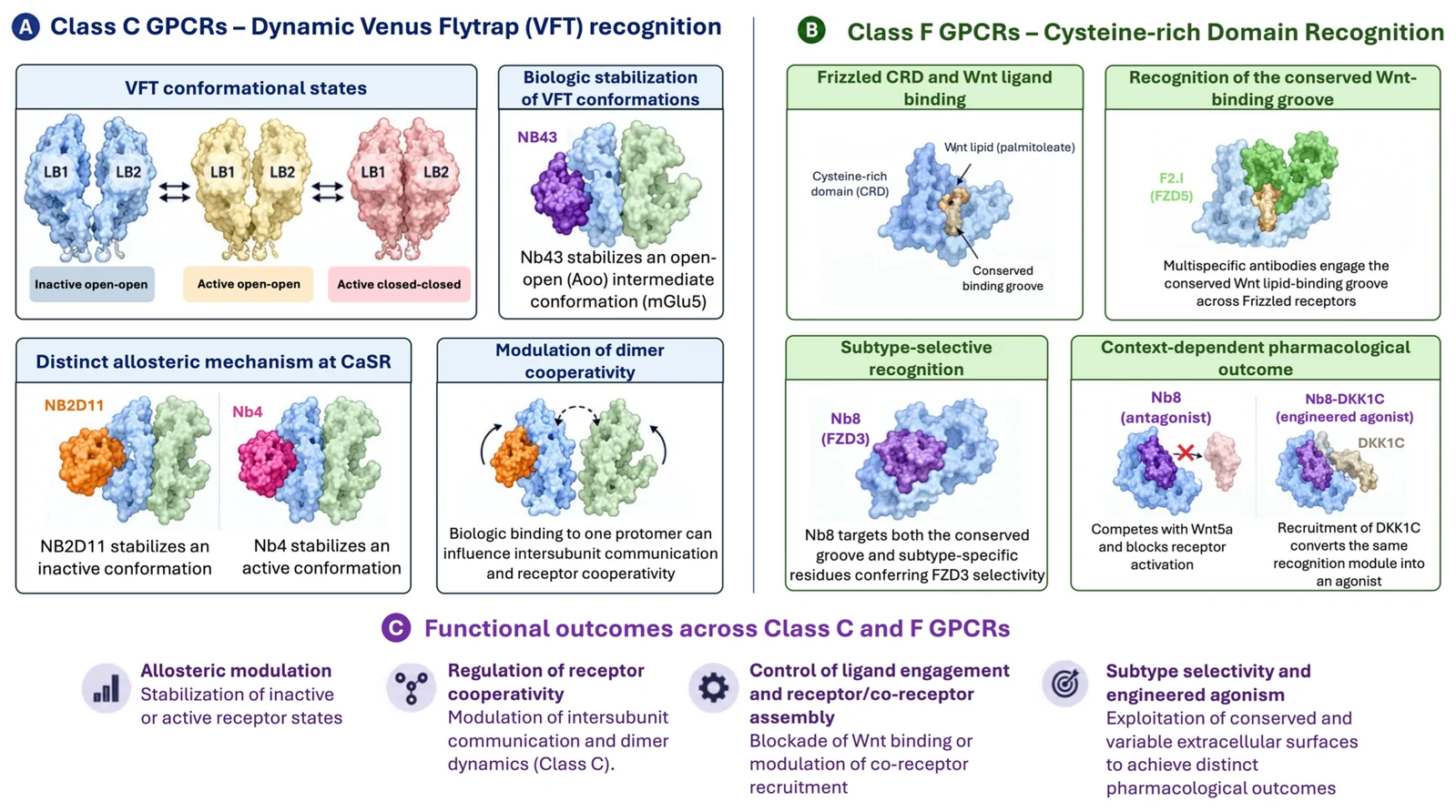
Structural principles governing biologic recognition in Class C and Class F GPCRs. (**A**) In Class C GPCRs, biologics exploit the conformational plasticity of Venus Flytrap (VFT) domains to stabilize distinct extracellular states and modulate receptor cooperativity. Nb43 stabilizes an active open-open (Aoo) intermediate conformation of metabotropic glutamate receptor subtype 5 (mGlu5), whereas the calcium-sensing receptor (CaSR)-directed nanobodies NB2D11 and Nb4 preferentially stabilize inactive and active extracellular conformations, respectively. Biologic stabilization of VFT conformations can additionally influence intersubunit communication and receptor cooperativity. (**B**) In Class F GPCRs, the cysteine-rich domain (CRD) provides both conserved and subtype-variable recognition surfaces. Multispecific antibodies targeting the conserved Wnt lipid-binding groove inhibit ligand engagement across Frizzled receptors, whereas Nb8 recognizes a subtype-selective surface of Frizzled Class Receptor 3 (FZD3). Fusion of Nb8 to the co-receptor-binding DKK1C module converts the same recognition element into an engineered agonist by promoting productive receptor–co-receptor assembly. (**C**) These mechanisms illustrate how the large and structurally organized extracellular domains of Class C and Class F GPCRs support allosteric modulation, regulation of receptor cooperativity, control of ligand engagement and receptor assembly, subtype-selective recognition, and engineered agonism. In panel (**A**), black bidirectional arrows indicate reversible transitions between VFT conformational states, whereas curved arrows represent intersubunit conformational communication and receptor cooperativity. In panel (**B**), the red cross indicates inhibition of Wnt5a–FZD3 interaction by Nb8, preventing receptor activation.

Class C and Class F GPCRs extend the relationship between extracellular architecture and biologic recognition beyond the modular ECD organization observed in Class B receptors (Figure 4).

In these families, large extracellular domains participate directly in ligand recognition, conformational regulation, receptor cooperativity, and higher-order assembly, creating recognition surfaces whose functional properties depend strongly on their structural state.

In Class C GPCRs, the dominant determinant is conformational plasticity. The VFT domains populate distinct inactive, intermediate, and active configurations, and biologics can selectively stabilize individual states along this landscape. Because these conformational changes are coupled across receptor protomers, extracellular recognition can additionally modify intersubunit communication and receptor cooperativity. The accessible epitope is therefore defined not only by extracellular architecture but also by the conformational state and quaternary organization of the receptor.

Class F receptors illustrate a complementary principle. The CRD provides a structurally conserved extracellular scaffold containing both a functional Wnt lipid-binding groove and more variable surrounding surfaces. These features support broad ligand-blocking antibodies as well as subtype-selective nanobodies. The functional consequence of CRD recognition can additionally be modified through biologic architecture, as illustrated by conversion of the FZD3-directed Nb8 targeting module from an antagonist into a component of a synthetic agonist through co-receptor recruitment.

Collectively, Class C and Class F GPCRs demonstrate that increasing extracellular architectural complexity expands biologic recognition along two dimensions: the number and organization of accessible interaction surfaces and the conformational or assembly states encoded by those surfaces. Large extracellular domains therefore provide not only expanded binding platforms but also dynamic regulatory structures through which biologics can control allosteric communication, receptor cooperativity, and higher-order assembly.

## 5. A Unified Structural Framework for Biologic Recognition Across GPCR Classes

The growing number of experimentally resolved GPCR–biologic complexes has revealed that biologic recognition cannot be explained solely by receptor sequence or pharmacological classification. Comparison across receptor classes instead identifies two interdependent structural variables that consistently shape biologic engagement: the extent, accessibility, and three-dimensional organization of receptor surfaces available for recognition, and the conformational landscape associated with these surfaces. The first defines where and how a biologic can engage the receptor, whereas the second determines how that interaction is coupled to receptor function and pharmacological outcome. A comparative overview of the structural determinants, recognition strategies, representative biologics, and pharmacological outcomes identified for each GPCR class is provided in Table 1.

To complement this class-level comparison, Table 2 summarizes the key interacting residues and structural regions identified in representative GPCR–biologic complexes, together with the associated conformational rearrangements and pharmacological consequences. This residue-level comparison highlights how distinct molecular interactions can stabilize specific receptor states and produce different functional outcomes, providing a more detailed structural basis for the recognition principles discussed throughout this Review.

These architectural constraints are particularly relevant for biologics because their molecular dimensions strongly influence the receptor surfaces that can be accessed. Conventional antibodies preferentially engage extended, solvent-exposed regions, whereas smaller biologic scaffolds such as nanobodies can additionally penetrate extracellular vestibules and intracellular cavities. As extracellular regions become more extensive and structurally organized across GPCR classes, the number and diversity of accessible interaction surfaces increase, expanding the range of recognition strategies available to biologic modalities [58,59].

This relationship is evident across the GPCR superfamily. The compact extracellular architecture of most Class A receptors restricts large biologics primarily to composite surface epitopes, while smaller nanobodies can access deeper orthosteric or intracellular cavities. Class B receptors provide larger extracellular domains that support extensive recognition interfaces and modular coupling between ligand binding and activation. In Classes C and F, highly structured extracellular domains further expand the accessible recognition landscape while introducing conformational, cooperative, and assembly-dependent determinants of biologic engagement.

Architectural accessibility alone, however, does not determine biologic function. A second universal determinant is the conformational landscape of the receptor surface being recognized. Biologics frequently engage structural elements whose organization changes during receptor activation, including extracellular loops, orthosteric vestibules, intracellular transducer-binding cavities, extracellular domains, VFT modules, and receptor assembly interfaces. Recognition of these functional structural elements couples molecular binding to the conformational transitions underlying receptor activation and signaling.

The pharmacological consequence of biologic binding therefore depends on the conformational state stabilized by the interaction. Direct competition with endogenous ligands represents only one mechanism of receptor modulation. Biologics can also restrict domain movements, stabilize inactive or active conformations, reshape allosteric communication pathways, alter receptor cooperativity, or promote productive receptor–co-receptor assembly. Consequently, similar receptor epitopes can support distinct pharmacological outcomes when different conformational states are stabilized, while structurally distinct binding sites can converge on comparable functional effects when they influence the same activation pathway.

An emerging extension of this framework concerns cryptic allosteric sites, which become transiently accessible through receptor conformational fluctuations and may remain undetected in static experimental structures. Molecular dynamics simulations have demonstrated that these transient pockets can contribute to GPCR subtype selectivity. In the M1 muscarinic acetylcholine receptor, a cryptic pocket preferentially sampled during receptor dynamics was shown to contribute to the selective recognition of positive allosteric modulators, with mutagenesis experiments confirming the functional relevance of pocket opening [60]. More recently, large-scale molecular dynamics analyses across the GPCR superfamily have revealed extensive local conformational fluctuations, transient allosteric pockets, and lipid-dependent lateral access pathways that are not consistently captured by individual experimental structures [61]. Although these findings have primarily informed small-molecule drug discovery, they also suggest potential opportunities for biologic engineering. In particular, conformationally selective nanobodies and other compact binding scaffolds may exploit transiently exposed extracellular surfaces or stabilize receptor states associated with cryptic-site formation. However, direct structural evidence for biologic engagement of cryptic GPCR allosteric pockets remains limited, and the steric accessibility of these sites must be considered. Integrating molecular dynamics, conformational mapping, and experimental structural characterization could therefore extend the identification of biologic-accessible epitopes and facilitate the development of next-generation GPCR-targeted modulators.

In this framework, epitope accessibility determines the opportunity for recognition, whereas conformational coupling determines the functional consequence of recognition.

These principles have direct implications for structure-guided biologic design. Epitope selection should consider not only sequence conservation but also surface accessibility, three-dimensional organization, and functional coupling to receptor activation. Large extracellular domains and composite interfaces provide opportunities for high-affinity and subtype-selective recognition, whereas conformationally dynamic regions offer the possibility of state-selective modulation. Structural characterization should therefore be integrated with functional validation to distinguish biologics that simply bind with high affinity from those capable of selectively reshaping receptor conformational equilibria and signaling output.

The clinical translation of GPCR-targeted biologics further illustrates the therapeutic relevance of distinct receptor recognition strategies (Table 3).

Approved monoclonal antibodies such as erenumab and mogamulizumab demonstrate the clinical applicability of extracellular receptor recognition, although their therapeutic effects arise from different mechanisms, including direct receptor antagonism and immune effector recruitment, respectively [16,21]. In parallel, peptide-based therapeutics targeting Class B GPCRs, including semaglutide and teriparatide, illustrate how the physiological ligand-binding architecture can be exploited to achieve clinically effective receptor activation [44,46]. Additional receptor-directed biologics have entered clinical investigation, extending the therapeutic landscape beyond established antibody and peptide modalities. These examples highlight the translational potential of GPCR-targeted biologics while emphasizing that clinical development remains unevenly distributed across receptor classes and molecular modalities.

The integration of cryo-EM, molecular dynamics, computational protein design, and artificial intelligence should increasingly enable these structural principles to be translated into predictive biologic engineering. Structural models can guide the identification of accessible functional surfaces, optimization of binding geometry, and design of biologics with tailored conformational and pharmacological properties. Emerging formats, including multispecific antibodies, fusion proteins, nanobody conjugates, and de novo designed binders, further expand the range of receptor architectures that can be exploited [59,68].

The emerging framework therefore extends beyond receptor classification. Across GPCR classes, biologic recognition is governed by the interplay between architectural accessibility and conformational plasticity. Receptor architecture defines the surfaces available for engagement, while receptor dynamics determine how binding is propagated through the signaling machinery. Increasing extracellular structural complexity expands both the number of accessible interaction surfaces and the conformational or assembly states that can be selectively targeted. Understanding these two dimensions provides a unified basis for interpreting existing GPCR–biologic complexes and for designing future biologics with controlled selectivity, efficacy, and signaling properties.

## 6. Conclusions

The comparative analysis of GPCR–biologic complexes across Classes A, B, C, and F identifies receptor surface accessibility and conformational dynamics as two interdependent determinants of biologic recognition and modulation. Specific structural examples illustrate how these principles translate into distinct pharmacological outcomes. In Class A receptors, the APJ-directed nanobodies JN241 and JN241-9 demonstrate how a minimal modification of the receptor-binding interface can convert antagonism into agonism through stabilization of different receptor conformations. In Class B receptors, engineered nanobody-peptide conjugates targeting PTH1R illustrate how the spatial relationship between extracellular recognition and transmembrane activation modules can be exploited to control signaling. In Class C receptors, conformationally selective nanobodies targeting mGlu5 and CaSR highlight the importance of extracellular domain dynamics, whereas FZD3-directed biologics demonstrate how receptor recognition can be combined with engineered co-receptor recruitment to alter signaling output. These examples establish that the pharmacological consequences of biologic engagement depend on both the structural accessibility of the target epitope and its coupling to receptor conformational transitions. Integrating these principles with experimental structural biology and computational protein engineering may support the development of GPCR-targeted biologics with improved selectivity and controlled functional properties.

## Figures and Tables

**Table 1:** Comparative structural framework for biologic recognition across G protein-coupled receptor (GPCR) classes.

GPCR Class	Defining Receptor Architecture	Principal Structural Determinant	Main Biologic Modalities	Recognition Strategies	Representative Examples	Pharmacological Outcome
Class A	Compact extracellular surface surrounding a conserved 7TM core	N-terminus, extracellular loops, orthosteric vestibule, intracellular transducer-binding cavity, extracellular conformational surfaces	Monoclonal antibodies, nanobodies, aptamers	Extracellular surface recognition; orthosteric pocket penetration; intracellular active-state stabilization; extracellular conformational recognition	Leronlimab-CCR5; mogamulizumab-CCR4; VUN701-ACKR3; NbE-μOR; JN241/JN241-9-APJ; Nb80-β2AR; Nb39-opioid receptors; Nb29-α1AAR	Receptor blockade, agonism, antagonism, inverse agonism, positive allosteric modulation, active-state stabilization, signaling bias
Class B	Large N-terminal extracellular domain functionally coupled to the 7TM bundle	Extended ECD surfaces, ECD-stalk-ECL coupling, two-domain ligand-binding architecture, and receptor–biologic geometry	Monoclonal antibodies, engineered peptide agonists, nanobody-peptide conjugates, bitopic biologics	ECD recognition; physiological two-domain binding; simultaneous ECD and TMD engagement	Erenumab-CGRPR; mAb1/mAb23-GCGR; semaglutide-GLP-1R; LA-PTH-PTH1R; tethered nanobody-PTH conjugates	Ligand blockade, receptor activation, prolonged signaling, improved potency and selectivity, signaling bias, geometry-dependent modulation
Class C	Obligate dimeric organization with dynamic Venus Flytrap domains	VFT conformational states, dimeric organization, intersubunit conformational coupling, and VFT-CRD-7TM communication	Nanobodies, antibodies, allosteric biologics	Conformation-selective VFT recognition; stabilization of inactive, intermediate, or active extracellular states; modulation of receptor cooperativity	Nb43-mGlu5; NB2D11-CaSR; Nb4-CaSR	Positive or negative allosteric modulation, conformational stabilization, and regulation of receptor cooperativity
Class F	Extracellular cysteine-rich domain linked to the 7TM core	Conserved Wnt lipid-binding groove, subtype-variable CRD surfaces, receptor and co-receptor assembly interfaces	Monoclonal antibodies, nanobodies, engineered Wnt surrogates	CRD recognition; blockade of Wnt engagement; subtype-selective targeting; modulation of receptor assembly	Multispecific anti-Frizzled antibodies; FZD3-directed Nb8; Nb8-DKK1C Wnt surrogate	Wnt antagonism, subtype-selective recognition, modulation of receptor assembly, and engineered agonism through co-receptor recruitment

**Table 2:** Comparative analysis of key molecular determinants, conformational changes, and functional consequences in representative GPCR–biologic complexes.

GPCR–Biologic Complex	GPCR Class	Key Interacting Residues or Structural Regions	Structural/Conformational Changes	Functional Consequences	Reference
ACKR3-VUN701	A	VUN701: Lys100, Arg103 (CDR3), His32 (CDR1); ACKR3: Asp275^6.58^, Glu290^7.27^, Asp179^4.61^, Phe285 ^ECL3^	Restriction of TM7 displacement (~5 Å) and stabilization of an inactive-like conformation	Competitive antagonism; subtype selectivity	[14]
μOR-NbE	A	NbE: Tyr106, Phe107, Tyr108 (CDR3); μOR: Lys209, Arg211, Gln212, Leu219^ECL2^, Lys303^6.58^, Glu310^ECL3^	Orthosteric pocket engagement with rearrangements of ECL2, ECL3, TM6 and TM7	Antagonism; subtype-selective recognition	[25]
APJ-JN241/JN241-9	A	JN241-9: inserted CDR3 Tyr; APJ: Tyr299^7.43^	Local remodeling of TM7 and the NPxxY-associated activation network	Antagonism-to-agonism conversion	[26]
β2AR-Nb80	A	Intracellular TM3/TM5/TM6/TM7 interface; Arg131^3.50^, Glu268^6.30^, Ile121^3.40^, Pro211^5.50^, Phe282^6.44^, Tyr326^7.53^	TM6 outward displacement (~11 Å); rearrangement of the ionic lock, PIF and NPxxY motifs	Active-state stabilization; increased agonist affinity	[57]
CGRPR-erenumab	B	CLR: Trp72, Ser117, Arg119; RAMP1: Trp74, Trp84; antibody: Tyr103, Tyr104, Tyr109, Tyr110 (CDR-H3)	Occupancy of the composite CLR-RAMP1 extracellular ligand-binding interface	CGRP receptor antagonism	[16,39]
GLP-1R-semaglutide	B	GLP-1R: Leu384^7.39^, Glu387^7.42^, Leu388^7.43^; semaglutide: His7, Aib8, Glu9, Thr11	Local remodeling of TM7 interactions while maintaining the two-domain peptide-binding mode	Receptor agonism	[44]
mGlu5-Nb43	C	VFT loops 345–357 and 365–375; Nb43 CDR2/CDR3	Stabilization of the active open-open (Aoo) intermediate	Positive allosteric modulation	[17]
CaSR-NB2D11/Nb4	C	VFT lower lobe and intersubunit interface; specific interacting residues not assigned in the comparative text	NB2D11 stabilizes inactive extracellular states; Nb4 favors active VFT conformations	Negative/positive allosteric modulation	[51]
FZD3-Nb8/Nb8-DKK1C	F	FZD3 CRD: Lys112, Met116; Nb8 recognition interface	Nb8 restricts CRD-associated interactions; Nb8-DKK1C promotes receptor–co-receptor assembly	Antagonism/engineered agonism	[55]

**Table 3:** Representative GPCR-targeted biologics approved for clinical use or investigated in clinical trials.

Biologic	GPCR Target (Class)	Modality	Mechanism of Action	Therapeutic Indication	Clinical Status *
Erenumab (Aimovig)	CGRP receptor, CLR-RAMP1 (B)	Human monoclonal antibody	Blocks CGRP receptor activation	Migraine prevention	FDA-approved [62]
Mogamulizumab (Poteligeo)	CCR4 (A)	Afucosylated monoclonal antibody	CCR4-directed immune cell depletion through ADCC	Mycosis fungoides; Sézary syndrome	FDA-approved [63]
Semaglutide (Ozempic/Wegovy)	GLP-1R (B)	Modified peptide agonist	GLP-1R activation	Type 2 diabetes; obesity and associated indications	FDA-approved [64]
Teriparatide (Forteo)	PTH1R (B)	Recombinant PTH(1–34) peptide	PTH1R agonism	Osteoporosis	FDA-approved [65]
Leronlimab (PRO 140)	CCR5 (A)	Humanized monoclonal antibody	CCR5 binding and inhibition of receptor-mediated interactions	Investigational oncology applications, including metastatic colorectal cancer	Phase II clinical investigation [66]
Denikitug (GS-1811)	CCR8 (A)	Afucosylated monoclonal antibody	CCR8-directed targeting of intratumoral regulatory T cells	Advanced solid tumors	Phase I clinical development [67]

* Regulatory approval and clinical development status were verified using official FDA documentation and ClinicalTrials.gov records, respectively, as of 13 October 2026. Clinical phases refer to the specific registered studies cited for investigational biologics.

## Data Availability

No new data were created or analyzed in this study. Data sharing is not applicable to this article.

## Abbreviations

The following abbreviations are used in this manuscript:

GPCR	G protein-coupled receptor
GRK	G protein-coupled receptor kinase
cryo-EM	cryo-electron microscopy
7TM	seven-transmembrane
ECL	extracellular loop
CDR	complementarity-determining region
CRS	chemokine recognition site
ADCC	antibody-dependent cellular cytotoxicity
NMR	nuclear magnetic resonance
TM	transmembrane helix
ICL	intracellular loop
Gαs	stimulatory G protein α subunit
Gs	stimulatory heterotrimeric G protein
PAM	positive allosteric modulator
RNA	ribonucleic acid
SELEX	systematic evolution of ligands by exponential enrichment
ECD	extracellular domain
CGRP	calcitonin gene-related peptide
CLR	calcitonin receptor-like receptor
RAMP	receptor activity-modifying protein
TMD	transmembrane domain
GLP-1	glucagon-like peptide-1
OEG	oligoethylene glycol
PTH	parathyroid hormone
cAMP	cyclic adenosine monophosphate
VFT	Venus Flytrap domain
CRD	cysteine-rich domain
LRP	low-density lipoprotein receptor-related protein
